# Supplementary material for: Development of a computer-aided design software for the quantitative evaluation of aesthetic damage
Source: PLoS One. 2019 Dec 18;14(12):e0226322. doi: 10.1371/journal.pone.0226322 (PMC6919621; doi:10.1371/journal.pone.0226322)
Supplement: S1 Code site — 1. (ZIP) [file pone.0226322.s001.zip › [EESC_jr][Projeto]Relatorio_07_21/reports/templates_zip/medialoot-lumino-admin-bootstrap-template.zip!/Lumino Admin Bootstrap Template/lumino/widgets.html]

Lumino - Widgets


Toggle navigation

LuminoAdmin

- User 
  - Profile
  - Settings
  - Logout

- Dashboard
- Widgets
- Charts
- Tables
- Forms
- Alerts & Panels
- Icons
- Dropdown
  - Sub Item 1
  - Sub Item 2
  - Sub Item 3
- Login Page

2. Icons

# Widgets

120

New Orders

52

Comments

24

New Users

25.2k

Page Views

Contact Form

Name

Your E-mail

Your message

Submit

Chat

- **John Doe** 32 mins ago

  Lorem ipsum dolor sit amet, consectetur adipiscing elit. Nulla ante turpis, rutrum ut ullamcorper sed, dapibus ac nunc. Vivamus luctus convallis mauris, eu gravida tortor aliquam ultricies.
- **Jane Doe** 6 mins ago

  Mauris dignissim porta enim, sed commodo sem blandit non. Ut scelerisque sapien eu mauris faucibus ultrices. Nulla ac odio nisl. Proin est metus, interdum scelerisque quam eu, eleifend pretium nunc. Suspendisse finibus auctor lectus, eu interdum sapien.
- **John Doe** 32 mins ago

  Lorem ipsum dolor sit amet, consectetur adipiscing elit. Nulla ante turpis, rutrum ut ullamcorper sed, dapibus ac nunc. Vivamus luctus convallis mauris, eu gravida tortor aliquam ultricies.

Send

Calendar

To-do List

- Make a plan for today
- Update Basecamp
- Send email to Jane
- Drink coffee
- Do some work
- Tidy up workspace

Add
